# Weight stigma and bias: A guide for pediatric clinicians

**DOI:** 10.1016/j.obpill.2023.100058

**Published:** 2023-03-20

**Authors:** Amy Braddock, Nancy T. Browne, Marcella Houser, Giselle Blair, Dominique R. Williams

**Affiliations:** a University of Missouri, 1 Hospital Drive, Columbia, MO, 65212, USA; b LSUHSC School of Medicine, Department of Pediatrics, 200 Henry Clay Ave., New Orleans, LA, 70118, USA; c The Ohio State University College of Medicine Center for Healthy Weight and Nutrition, Nationwide Children's Hospital, 700 Children's Drive LA, Suite 5F, Columbus, OH, 43215, USA; d East Carolina University, USA

**Keywords:** Childhood obesity, Obesity, Pediatrics, Weight bias, Weight stigma

## Abstract

**Introduction:**

Many children and adolescents with obesity experience weight stigma and bias, which can have detrimental mental health, medical, and social consequences. Weight stigma in the healthcare setting threatens the therapeutic relationship between health care providers and their pediatric patients and families.

**Methods:**

Data supporting this guidance were derived from cited references.

**Results:**

Based upon referenced citations, this review offers 7 best practices for pediatric providers to work to reduce weight stigma including: assess for personal weight bias, improve communication, provide a welcoming clinic environment, seek out additional training and informative experiences, evaluate the messaging and culture of the organization, screen for trauma and bullying, and enlist the help of board-certified obesity medicine specialists.

**Conclusions:**

Providers have an important role in mitigating the harmful effects of weight stigma. It is our hope these recommendations, as well as the other resources provided, will help providers to begin to address their own individual weight biases, as well as the institutional weight biases where we care for patients.

## 1 Background

With pediatric obesity rates continuing to rise, it is important for health care providers to be aware of weight bias and stigma affecting children and adolescents and proactively develop strategies to address it. Weight stigmatization, defined as a societal devaluation projected on individuals with obesity, can include implicit or explicit bias, discrimination, teasing, bullying and victimization, all of which can contribute to adverse health outcomes [1]. The self-devaluation experienced by some children and adolescents with obesity is compounded by many external sources of weight stigmatization including peers, family, educators, traditional print and social media, and health care providers. Obesity-related weight bias is so ubiquitous, it has been documented in toddlers as young as 32 months with preferences for looking at average-weight figures over those with increased weight [2]. The harmful impacts of weight stigma occur in all races, ages [3], family socioeconomic status (SES), levels of academic achievement [4], gender identity and sexual orientation [5].

Health care providers are identified as one of the most frequent perpetrators of weight stigmatization experienced by patients. More than two-thirds of women with overweight or obesity self-reported experiencing stigmatization about their weight from their doctors [6]. In a 2021 study by Phelan et al., patients with obesity experience less patient-centered care and more negative normative attitudes [7]. Health care providers and trainees self-report high levels of bias against patients with obesity, continuing stereotypes that these patients are lazy, lack self-control and are less intelligent [3]. Implicit and explicit weight biases were held by 75% of faculty and 66% of medical students respectively. Higher levels of bias were observed among medical students with lower body mass index (BMI), male sex, and an interest in subspecialties (as opposed to primary care) [8]. The current culture of medical education was founded on the simplistic “eat less, move more” mentality of addressing obesity and likely contributes to weight bias and stigma during medical training. Health care providers who serve as faculty attendings often lack specific training in obesity medicine and may model use of discriminatory or disparaging obesity language due to lack of understanding of the complexity of this disease [1,7].

In addition to having bias against children and adolescents with obesity, health care providers and care teams may also exhibit weight stigma by association, where they blame or shame caregivers for the child's increased weight [9]. In a randomized online survey, 1862 participants were randomly assigned to view a picture of a parent-child dyad with or without obesity. Compared to parents and children without obesity, participants rated parents *with obesity* and parents to *children with obesity* as being less effective caregivers [10].

Obesity in children is associated with cardiometabolic and mental health comorbidities [11]. Additionally, severe obesity is associated with reduced health related quality of life (QOL), with QOL scores in children and adolescents with severe obesity similar to those in children and adolescents diagnosed with cancer [12]. These harms are exacerbated by weight stigmatization, as they can impede therapeutic relationships between providers and patients or families. When parents were asked how they would respond to providers who exhibited weight stigmatization toward their children, 35% reported they would seek a new doctor and 24% would avoid future medical appointments altogether [13]. These barriers to seeking health care compromise the management of other chronic diseases, as patients avoid seeking care because they are concerned their health issue will be attributed to weight alone. Physicians also spend less time counseling and are less likely to perform preventive health screenings in patients with obesity [14]. All of these factors impair the provider's ability to deliver effective, patient-centered care, as well as the development of therapeutic relationships, and may contribute to increased weight and other chronic diseases in this population [3].

The belief that “telling it like it is” will motivate an individual to lose weight is incorrect and harmful and perpetuates weight stigma [15]. Rather, the bidirectional nature of weight stigma suggests that not only is a higher BMI associated with higher likelihood of experiencing weight stigma, but weight stigma is associated with increased BMI [16]. Weight stigma is a psychosocial contributor to stress associated with weight gain [5,17]. A large body of evidence supports the harmful mental health impacts of weight stigmatization on children and adolescents. For example, repeated exposure to weight-based teasing, bullying, or victimization can lead to weight bias internalization – or internalized weight stigma. Internalized weight bias, or when a person attributes negative beliefs about their weight to themselves, causing a belief in weight-based stereotypes, results in lower self-esteem in children [18]. Weight bias internalization is also associated with physical inactivity and avoidance, sleep disturbance, stress and disordered eating behaviors (i.e. binge and emotional eating) [5,17]. Moreover, the detrimental effect of weight stigmatization on youth mental health [15] can include social isolation [3], depression, and suicide ideation [19].

Additionally, weight stigmatization by peers can include teasing, bullying, or cyber bullying, with 71% of adolescents who are seeking weight loss treatment reporting being bullied about their weight in the past year [20]. Students can also suffer academic underachievement due to unfair grading by teachers or social isolation from other students [21,22]. Additionally, when students experience stereotype threat (where students feel that they are at risk of upholding weight-related stereotypes), weight stigmatization can further impair academic achievement by reducing working memory through impaired executive function [23].

The effects of weight bias and stigma are likely intensified in those belonging to multiple socially marginalized groups [24,25]. In study of sexual and gender minority adolescents, over half of this nationally surveyed cohort experience weight-based victimization by their family members (55.4%) or peers (50.4%); whereas, the rate of victimization was much lower (40%) in those who did not identify as sexual or gender minority [24]. Compared to *cis*-gender youth, transgender and gender diverse (TGD) youth have significantly higher rates of mood disturbance, substance abuse, self-injurious behaviors and are exposed to more violence and victimization, including child abuse [25]. As a result, TGD youth are especially vulnerable to patterns of abnormal weight gain due to higher rates of trauma and chronic stress [25].

The Minority Stress Model “posits that stigmatized and minoritized groups endure excess psychological and physical stress due to their perceived social position” and “accounts for gender identity, sexual orientation, weight, race, ethnicity as well as income and other socioeconomic circumstances” [25]. In the context of obesity, stress “results from stigma, social isolation and rejection …” [25]. Obesity disproportionately affects marginalized and racially minoritized groups. The prevalence of obesity in non-Hispanic Black (25.1%) and Mexican American (24.9%) youth is almost 75% higher than non-Hispanic White (14.7%) youth; and more 200% higher in American Indian/Alaska Native youth (29.7%). Moreover, negative social determinants of health like food insecurity harm groups already at risk for obesity, such as those with low income or lower education.

In study of sexual and gender minority adolescents (n = 9838), over half of this nationally surveyed cohort experienced weight-based victimization by their family members (55.4%) or peers (50.4%); whereas the rate of victimization was much lower (40%) in those who did not identify as sexual or gender minority [24]. Further analysis also revealed that transgender boys (64.4%) and assigned female at birth (AFAB) non-binary youth (61.6%) experienced significantly higher rates of weight-based teasing from family members, compared to cisgender boys (43.7%) and girls (56.8%) [26]. Furthermore, almost 40% of the cohort had overweight or obesity (BMI >85th percentile). In linear regression, BMI percentile and gender identity were significantly linked with more dieting, binge eating and weight control behaviors (healthy and unhealthy) compared to cisgender boys; the exception was transgender girls and assigned male at birth (AMAB) non-binary youth. Likewise, through logistic regression, BMI percentile and gender identity were also significantly linked to physical inactivity and more avoidance of exercise – except in AMAB non-binary youth. In a study of youth aged 14–18 years old (n = 2020), compared to cisgender youth, TGD youth have significantly higher odds ratio (OR) of suicidal ideation (OR = 2.2), suicide attempt (OR = 1.65) and non-suicidal self-injurious behavior (OR = 2.88) [27].

Although TGD youth, those from racially minoritized and marginalized groups, as well as those experiencing negative social determinants of health are vulnerable to the effects of trauma, chronic stress, and social rejection, the data are lacking to adequately describe their intersections with weight bias [25]. Nonetheless, there is compelling data to be considerate of the individuality of each patient in order to deliver holistic care [25].

## 2 Best practices for clinicians to reduce bias, stigma, and weight based victimization of children and adolescents with obesity

**Fig. 1:**
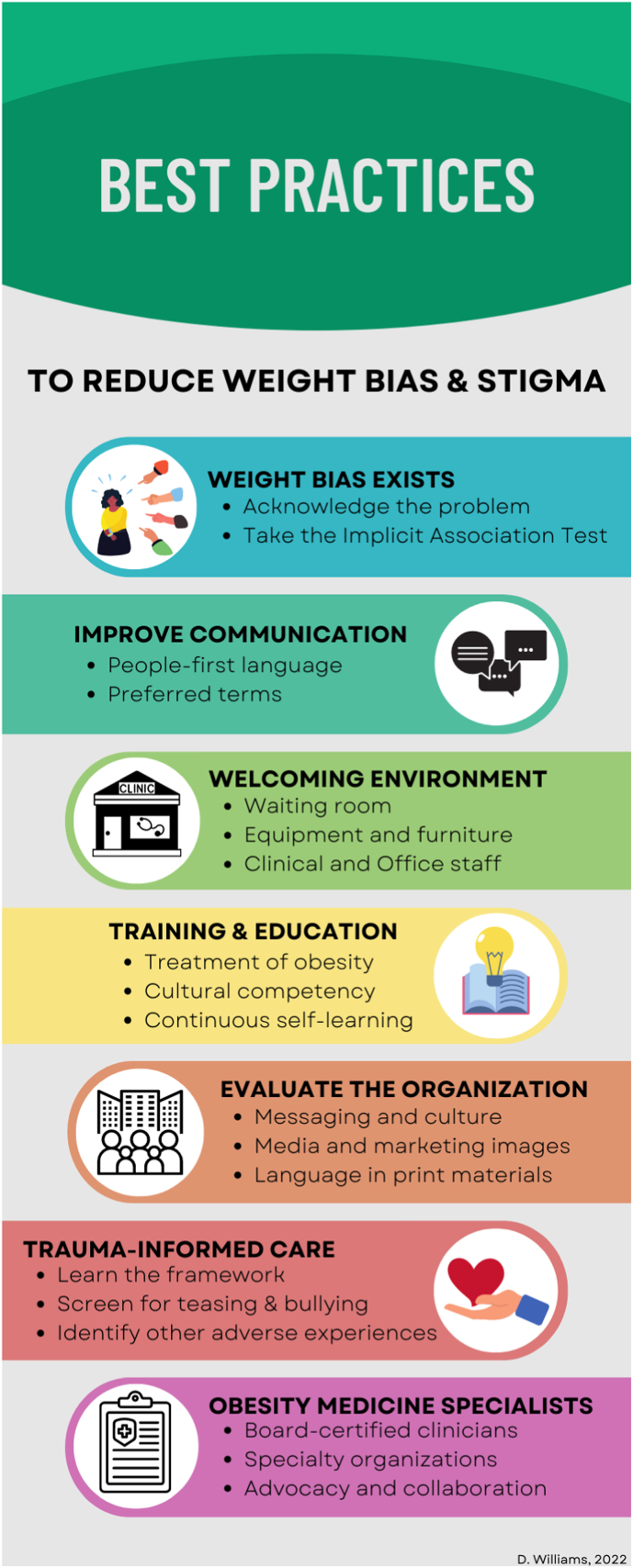
Best practices for clinicians to reduce bias, stigma, and weight based victimization of children and adolescents with obesity.

Encounters with health care providers are an opportunity to mitigate these effects and can help build resilience in the child and confidence in the caregivers. With the goal of reducing weight stigmatization and in accordance with recommendations from obesity medicine and pediatric organizations, we offer the following best practices for health care clinicians and organizations [1,3,19,[28], [29], [30]]. These recommendations are summarized in Fig. 1. Additional recommendations for providers to reduce weight stigma are listed in Table 1. These recommendations are intended to supplement resources.

1. **Assess for personal weight bias:** Weight bias is prevalent in our culture. With acknowledgement comes awareness that can inform our thoughts and behaviors to reduce bias. The Implicit Association Test [IAT] is a validated assessment tool to assess weight bias [31,32] (and other biases) by measuring the strength of associations between concepts and stereotypes. The goal of use of the IAT is self-awareness and thoughtful consideration of our thoughts, words, and actions.
2. **Improve communication**:
  a. **Use person first language**: Use of person first language should extend to all medical conditions and diseases but is especially important for the disease of obesity. Terms like “obese” or “fat” are particularly stigmatizing. “Person with obesity” is the preferred terminology. Make every attempt to use person first language in verbal and written communications with the patient, with scientific writing [33], as well as during teaching rounds and medical education [34]. Modeling this behavior as a trusted clinician, educator and colleague makes a strong statement and encourages others to do the same. Pediatric clinicians and support staff may be one of the few groups providing tangible support to an individual child vulnerable to weight stigmatization.
  b. **Words matter:** In addition to asking permission to talk about weight, take an additional step and ask patients about their preferred terms [35]. Children and adolescents dislike terms like “’[morbidly or extremely] obese,” “fat,” or “large” as they trigger feelings of guilt and shame [3,19,36]. In contrast, there is some variability in preferred terms and emotional response depending on sex, sexual orientation, racial/ethnic background [36]. In a study of preferred weight terminology in youth ages 10–17 years (n = 2032), Puhl et al. (2022) learned that the term “fat” produced significantly more negative reactions in white youth compared to Black/African American youth [36]. On the other hand, the term “curvy” was more preferred by girls, sexual minority, and Hispanic/Latinx youth compared to boys, heterosexual and white counterparts. Among all groups, “healthy weight” and “normal weight” are generally the most preferred terms. These nuanced differences in preferred terms demonstrates the importance of communicating with patients to identify their desired terms [35,37]. Table 1 provides additional guidance
3. **Provide a welcoming clinic environment**: An organization that has appropriate resources for patients with obesity validates the level of concern for a patient's safety, comfort, and physical and psychosocial needs [38]. In the waiting room and exam rooms, use furniture that is safe and comfortable to all body habitus weights, and feature appropriate reading materials that respectfully depict people with obesity and avoid glorifying thinness as the standard of beauty. When triaging the patient, ensure privacy when weights are obtained (and weigh with permission) and use equipment that is validated for patients with higher weights and different sizes (e.g. blood pressure cuffs, scales). In the exam room, offer gowns/robes that are appropriately sized to provide modesty and coverage [39].
4. **Seek out additional training and informative experiences**: These trainings can help providers on several fronts including learning to reframe obesity as a chronic disease and not a personal weakness or lifestyle choice [40], and to understand the biochemical complexity [41,42] that contributes to obesity and hinders weight loss. Look for educational offerings that focus on understanding external factors including genetics [28], social determinants of health and historical trauma that lead to stress, affect growth and development, and contribute to chronic diseases like obesity [43]. Cultural competency training can help providers understand the interplay between their culture, personal beliefs and experiences with the responses and experiences of their patients [44]. Cultural competency training can help to inform communication and behaviors that may affect therapeutic disease management. Other ways to mitigate bias include looking for shared experiences with patients with obesity; look for movies or TV shows that counter stereotypical depictions of people with obesity and portray characters that are intelligent, hard-working, and motivated [45]. These training should begin in medical school, continue through residency, and also occur regularly for practicing providers [3], to help further mitigate weight bias.
5. **Evaluate the messaging and culture of the organization**: Respectful and empathetic care for patients with obesity must be planned and intentional with ongoing updates and evaluation [46]. A clinician's best efforts may be undermined by biased messaging within the organization. Who is portrayed on the organization's website? What words are used to describe the weight management clinic (if there is one). Is person first language and appropriate terminology used? Are images of individuals with obesity respectful? It is important to be aware of the messaging and imaging portrayed of patients with and without obesity on advertising and media. Guidelines are available for internal organizational media and for advocating for appropriate media coverage in the community [47]. Inappropriate images and media (internal and external) are a frequent and unchecked contributor to weight bias. All members of the organization have a responsibility to identify examples and collaborate to make changes when inappropriate materials are encountered.
6. **Screen for trauma and bullying:** Use trauma informed care (TIC) principles [43] with every appropriate clinical encounter to assess weight stigma and bullying, [[48], [49], [50], [51], [52]], and in the assessment for weight-based victimization or other forms of trauma. Table 1 [[48], [49], [50], [51], [52], [53]] The TIC framework of realize, recognize, respond, and resist (re-traumatization) is particularly relevant to obesity care as research estimates that 50–66% of youth with obesity report some form of weight-related teasing, bullying and discrimination that contributes to ongoing stress and vulnerability to mood disturbance and chronic disease [53].
7. **Enlist the help of board-certified obesity medicine specialists:** Board certification in Obesity Medicine is available for providers wanting to enhance their knowledge and skills in managing obesity. The American Board of Obesity Medicine (ABOM) offers physicians certification (ABOM diplomate), signifying specialized knowledge in the practice of obesity medicine and achieving competency in obesity care. Similarly, the Obesity Medicine Association (OMA) offers Nurse Practitioners (NP) and Physician Assistants (PA) the Certificate of Advanced Practice to demonstrate extensive knowledge of evidence-based obesity medicine treatment approaches. The Obesity Society also offers a practice management and leadership training for PAs and NPs [54]. In addition to being a referral resource for patients with obesity, these professionals can also be strong advocates for this population, which can help reduce institutional weight stigma. OMA, TOS, and other ABOM-approved organizations all offer Continuing Medical Education (CME) for interested clinicians looking for in-depth, evidence-based educational content to better inform the care of children and adolescents with obesity.
8. **Additional Resources**:
  a. The Obesity Medicine Association https://obesitymedicine.org/what-is-obesity/
  b. Rebecca Puhl, Rudd Center for Food Policy and Obesity, University of Connecticut, Addressing Weight Bias: Resources and Tools from the Rudd Center http://www.uconnruddcenter.org/weight-bias-stigma
  c. Balanced View: Addressing Weight Bias and Stigma in Health Care https://balancedviewbc.ca (5 Module course)
  d. *Joint International Consensus Statement for Ending Stigma of Obesity* [28] : this Expert Panel developed an open access Pledge and Consensus Statement (Table 2), which can be posted in clinic spaces as a visual reminder to patients, providers, and staff of a safe haven clinic.

**Table 2:** **Consensus statements on the stigma of obesity: recommendations Copyright@2020** Rubino F, Puhl RM, Cummings DE et al. Joint international consensus statement for ending stigma of obesity. *Nat Med*. 2020;26(4):485–497. https://doi.org/10.1038/s41591-020-0803-x This Table is reproduced exactly from this open access article distributed under the terms of the Creative Commons Attribution License.

General	Weight-based stigma and obesity discrimination should not be tolerated in education, healthcare, or public-policy sectors
	Explaining the gap between scientific evidence and the conventional narrative of obesity built around unproven assumptions and misconceptions may help reduce weight bias and alleviate its numerous harmful effects.
	The conventional narrative of obesity built around unproven assumptions of personal responsibility, and misconceptions about the causes and remedies of obesity causes harm to individuals and to society. Media, policy makers, educators, HCPs, academic Institutions, public health agencies, and government must ensure that the messages and narrative of obesity are free from stigma and congruent with modern scientific evidence.
	Obesity should be recognized and treated as a chronic disease in healthcare and policy sectors.
**Media**	We call on the media to produce fair, accurate, and non-stigmatizing portrayals of obesity. A commitment from the media is needed to shift the narrative around obesity.
**Healthcare and education of HCPs**	Academic institutions, professional bodies, and regulatory agencies must ensure that formal teaching on the causes, mechanisms, and treatments of obesity are incorporated into standard curricula for medical trainees, and other Health care providers.
	Health care providers specialized in treating obesity should provide evidence of stigma-free practice skills. Professional bodies should encourage, facilitate, and develop methods to certify knowledge of stigma and its effects, along with stigma free skills and practices.
	Given the prevalence of obesity and obesity-related diseases, appropriate infrastructure for the care and management of people with obesity, including severe obesity, must be standard requirement for accreditation of medical facilities and hospitals.
**Public Health**	Public health practices and messages should not use stigmatizing approaches to promote anti-obesity campaigns. These practices are objectively harmful and should be banned.
	Public health authorities should identify and reverse policies that promote weight-based stigma, while increasing scientific rigor in obesity-related public policy.
**Research**	Research in obesity and type 2 diabetes should receive appropriate public funding, commensurate to their prevalence and impact on human health and society
**Policies and Regulation**	There should be strong and clear policies to prohibit weight-based discrimination
	Policies and legislation to prohibit weight discrimination are an important and timely priority to reduce or eliminate weight-based inequities.

**Table 1:** Additional Practical guidance to reduce weight stigma in the clinic.

Patient Specific	
**Communication**	Acknowledge and discuss obesity as a chronic, complex disease with multiple contributors - “There are a lot of things that affect a person's health and weight” - “I am concerned about your total health. Weight is just one part of it. Is it okay if we talk about?”
	Review the broad range of contributors to obesity beyond increased calories and decreased activity - “Things like genetics, stress [trauma], sleep, and even medications can affect how our bodies use energy” - “There's a lot we're still learning about why some people have normal weight and others don't. What we do know is that it is not your fault. It's not because of lack of willpower or lack of motivation.”
	Inquire about how increased weight has affected their daily life, social interactions, schooling and extracurricular activities - “What has stressed you out recently? How have you been dealing with it?”
	Use reflective listening and summarization to validate the patient's concerns and determine next steps - “It sounds like you are worried about […] because its causing […]. A lot of people are concerned about […]. Where does that leave us?
	Identify negative speech during patient interviews and use it as a teaching moment for family and patient to change. - “When we talk about weight, what words would you like us to use? What words do you want us to avoid?”
**Assessment**	Use thorough clinical evaluation. Screen for and treat obesity-related conditions. Avoid over attributing weight or physical conditioning as reason for musculoskeletal pain or other complaints.
	Do not assume schools will be able to successfully address weight-based bullying on their own. Screen for mood disturbance and make referrals to community mental health resources
	Connect patients and families who are experiencing bullying with trained advocates or agencies who help families navigate the school system and are knowledgeable about student rights
**Treatment**	Present the full spectrum of treatment that corresponds with the patient's medical history and acuity, including intensive lifestyle modification (nutrition, physical activity), behavioral therapy, anti-obesity and adjunctive medications, and metabolic-bariatric surgery [53]
	Review the evidence-base with patients and families that refutes the idea that bariatric surgery is the “easy way out.”
	Implement Principles of Trauma Informed Care - **Realize** that trauma exists and can have both short- and long-term effects on the health of patients - **Recognize** and identify trauma by screening for adverse childhood experiences, weight-based victimization, mood disturbance - **Respond** with support of policies, practices and clinical workflows that support all patients, including taking universal precautions that all patients have experienced or are experiencing some type of trauma - **Resist Re-traumatizing** by reviewing the medical record, communicating with staff and coordinating care, and avoiding stigmatizing language

**Clinic Specific**	

	Develop a strategy to address the intersectionality of multiple biases propagated by society, such as gender diverse youth with obesity
	Utilize patient surveys and patient experience boards to allow patients and families to share experiences of weight bias.
	Attrition can result from a stigmatizing experience from anyone they encounter during their clinical visit or from organizational messaging. Try to obtain feedback through patient questionnaires of their reason for not attending clinic visits
	While negative billing codes are difficult to totally avoid, be intentional with documentation and try to avoid use of the terms “morbid obesity” or “obesity due to excess calories”. Use Z code descriptors to accompany E codes, and discuss system limitations with patients.

Abbreviation: ICD = International Classification of Diseases.

## 3 Conclusion

Although not an exhaustive list, it is our hope that these practical suggestions and actionable recommendations will better equip clinicians who care for children and adolescents with obesity to form therapeutic alliances, reduce weight bias and stigma, and improve patient experiences and outcomes. Media, family and friends, environmental factors and social drivers of health, all contribute to weight stigmatization, making it difficult to eradicate. Like other biases, awareness of its presence and making an effort to reduce its harmful effects, is the first step to addressing weight stigma of children and adolescents.

## Disclosures

No applicable disclosures.

## Credit authorship

The concept of the submission and first draft was by ASB. NTB, MH, GB, DRW contributed to additional drafts, reviewed, edited, and approved the final submission and publication.

## Ethical review

This submission represent original work and we have appropriately cited included works. This manuscript is being submitted only to *Obesity Pillars*. It will not be submitted elsewhere while under consideration, it has not been published elsewhere, and, should it be published in *Obesity Pillars*, it will not be published elsewhere without permission of the editors. All authors are responsible for the content of this review, and have participated in the concept, drafting or revising of the manuscript, and have approved the manuscript as submitted.

## Source of funding

This research did not receive any specific grant from funding agencies in the public, commercial, or not-for-profit sectors.

## Declaration of competing interest

All authors have no conflict of interest.
